# Mitigative effect of caffeine against diclofenac-induced hepato-renal damage and chromosomal aberrations in male albino rats

**DOI:** 10.1186/s12906-022-03802-y

**Published:** 2022-12-08

**Authors:** Mai M. Anwar, Ibrahim M. Ibrahim Laila

**Affiliations:** 1 Department of Biochemistry, National Organization for Drug Control and Research (NODCAR)/Egyptian Drug Authority (EDA), Cairo, Egypt; 2 National Organization for Drug Control and Research (NODCAR)/Egyptian Drug Authority (EDA), Cairo, Egypt; 3 Department of Biotechnology & Molecular drug evaluation, National Organization for Drug Control and Research (NODCAR)/Egyptian Drug Authority (EDA), Cairo, Egypt

**Keywords:** Caffeine, Diclofenac, NSAID, Antioxidant, DNA damage, Liver, Kidney

## Abstract

**Background:**

Among the most commonly consumed non-steroidal anti-inflammatory drugs (NSAID) is Diclofenac (Dic), especially in low-income countries due to its high efficiency and affordable price. However, the continuous administration of Diclofenac may induce toxic effects on various body organs including the liver and kidney. Caffeine (Caf) (1,3,7-trimethylxanthine) is a pharmacologically active alkaloid type with antioxidant and anti-inflammatory actions.

**Aim:**

The current study aims to evaluate the ameliorative effect of Caffeine against Dic-induced hepato-renal toxicity and damage.

**Methods:**

Twenty-four male albino rats type were assigned randomly into four groups (*n* = 6): (Group 1): Control group, (Group 2): Six male rats were exposed to Dic 10 mg/kg intraperitoneally (I.P) for 28 days, (Group 3): Six male rats were exposed to Caf (15 mg/kg orally) for 28 days; (Groups 4): Six male rats were exposed to Dic (10 mg/kg, i.p) + Caf (15 mg/kg, orally) for 28 days. Histopathological study and various biological parameters were estimated among the four groups including hemoglobin (Hb%) red blood cells (RBCs), Hematocrit (HT%), total leucocyte count (WBCs), lipid peroxidation (LPO), glutathione peroxidase (GPx), alanine aminotransferase (ALT), aspartate aminotransferase (AST), urea, creatinine, tumor necrosis factor-α (TNF-α), and nitric oxide (NO).

**Results:**

The administration of Diclofenac resulted in significant deteriorations in the histopathological findings and estimated biological parameters. Whereas, daily Caffeine administration ameliorated Diclofenac-induced toxicity in the kidney and liver by three mechanisms including antioxidant, anti-inflammatory, and DNA damage inhibition.

**Conclusion:**

The current study demonstrated the promising ameliorative and protective effects of Caffeine against Diclofenac-induced hepatic and renal injury.

## Introduction

NSAIDs are the type of the most commonly used drug as an analgesic, anti-inflammatory, and for the treatment of rheumatoid and osteoarthritis [1]. It was previously reported that NSAIDs directly inhibit cyclooxygenase-1 (COX-1) and cyclooxygenase-2 (COX-2) enzymes activity and thereby suppress the release of thromboxane and prostaglandin [2, 3]. Prolonged and misused chronic intake of NSAIDs may lead to undesirable drawbacks including neurotoxicity, nephrotoxicity, hepatotoxicity, cardiovascular diseases, and gastrointestinal injury [4–6]. Diclofenac (2-[(2,6-diclorophenyl)amino]phenyl acetate) is the most abundant and widely used NSAID phenylacetic acid derivative for its wide actions including antipyretic, anti-inflammatory, and pain relief [7]. The reason why Dic administration may results in renal damage is mainly due to reduced renal blood flow resulting in ischemia and necrosis along with elevated oxidative stress and inflammatory cytokines release [8]. Meanwhile, Dic-induced liver damage may also be related to inflammation, oxidative stress, and cytochrome P450 activation [9, 10].

Both the liver and kidney play vital roles in the elimination process of wastes produced in all living organisms. Thereby, any damage in both organs results in metabolic dysfunctions and the accumulation of toxins in the body leading to systematic toxicity, and atrophy [10, 11]. In addition to filtering unnecessary products from the blood, kidneys mainly maintain electrolytes/water balance, control the secretion of erythropoietin to regulate hematopoiesis, and modulate controlled blood pressure [12, 13]. Additionally, kidneys also regulate vascular tone and sodium level, by maintaining prostaglandins secretion in order to keep a balanced renin-angiotensin system [12, 13]. By reinforcing renin secretion, prostaglandins increase potassium secretion and prevent tubular reabsorption of sodium. Since the main function of prostaglandins secretion is to maintain normal kidneys’ glomerular filtration rate (GFR), their inhibition can be a drawback of excessive daily use of diclofenac leading to abnormal renal functions and chronic kidney diseases (CKD) on the long-term [14]. Diclofenac may induce renal injury by targeting the kidney’s mitochondria leading to the reactive oxygen species (ROS) over production, apoptosis and DNA lesions [15]. Meanwhile, regarding the mechanism of diclofenac-induced chronic liver diseases (CLDs) is idiosyncratic where excessive diclofenac is metabolized by multiple cytochrome P-450 enzymes in hepatocyte resulting in glutathione (GSH) conjugation, irreversible mitochondrial dysfunction and organ severe damage [16]. Chronic kidney and liver damages can also be related to severe cellular damage induced by exaggerated release of ROS and oxidative stress including hydroxyl radicle resulting in severe activated inflammatory responses [17]. These activated inflammatory responses mediate the release of nuclear factor-kappa B (NF- B), tumor necrosis factor-alpha (TNF-α), NO, and interleukin 6 (IL-6) [18].

The absence of potential compounds that can alleviate and protect vital organs from any damage is a major problem, especially in conventional medicine. Thereby it is extremely crucial to find out natural compounds that may protect our vital organs from induced damage and cytotoxicity due to daily misused consumed drugs [19]. Caffeine is a natural methylxanthines compound commonly found in beverages and coffee. Thus, caffeine has been recently studied for its various biochemical and physiological effects, including anti-inflammatory effects and antioxidant actions [20]. It was reported previously that Caffeine administration may exert hepatoprotective and nephroprotective effects by maintaining normal AST, ALT, creatinine, and urea [21, 22]. Some studies attributed these protective effects of Caffeine due to its anti-inflammatory and antioxidant actions by scavenging reactive oxygen species (ROS) such as hydroxyl radical (OH) along with decreasing lipid peroxidation and NO [23]. Several studies have reported the protective effect of daily caffeine consumption on liver and kidney against induced oxidative stress and activated inflammatory responses [24, 25]. Ruhl and Everhart [24, 26] reported that higher caffeine consumption hinders elevated alanine aminotransferase (ALT) and aspartate aminotransferase (AST) as a marker of liver injury resulting in decreased CLDs. On the other hand, several studies have reported a renal protective effect of caffeine consumption against CKDs by increasing the GFR and maintaining the renin-angiotensin system [25, 27].

The main aim of the current study was to assess whether Dic administration as a type of analgesic OTC drug may exert severe hepato-renal toxicity represented by biological changes, and histopathological damage. The study was also designed to evaluate the protective efficiency of daily Caffeine administration on hepatic and renal tissues against the induced damage by diclofenac. Our findings reported that caffeine may be considered as a protective agent against hepatic and renal toxicity by attenuating inflammatory responses and oxidative stress.

## Materials and methods

### Chemicals

Diclofenac sodium (Dic) was obtained from Novartis Pharmaceutical Company, Cairo, Egypt. Meanwhile, Caffeine was purchased from sigma Aldrich company (St. Louis, MO) (CAS Number: 58-08-2). Thiobarbituric acid (TBA) was purchased from Fluka Chemical company, and trichloroacetic acid (TCA) was purchased from Merck, USA. While Dinitrophenylhydrazine (DNPH) and 5-5-dithiobis-2-nitrobenzoic acid (DTNB) were obtained from Sigma chemicals, USA.

### Animals

All the conducted experimental procedures of the current study were initially approved by the Animal Research and Ethical committee of National organization of Drug Control and Research (NODCAR) approval number (NODCAR/II/53/2022) guided by the 3Rs principles (refine, reduce and replace). Twenty-four male Wistar Albino rats weighing from 200 to 250 gm (5–7 weeks old) were obtained from the animal facility house of (NODCAR, Cairo, Egypt). Rats were housed in the maintained conditions including 12 h. light/dark cycle, controlled temperature 22 ± 1℃ and with complete free access to food and water ad libitum. More importantly, the order of treatments assigned for each rat in the experimental design was equally balanced and onset period effects was considered along with rats body weight to avoid confounding effects and experimental error in the statistical study.

### Study design

The twenty-four male Wistar Albino rats were assigned randomly and equally divided into four Groups (*n* = 6 per group). (Group 1): Ctrl group received normal saline intraperitoneally and a daily 1 mL distilled water by oral gavage to provide the same conditions as the other three groups, (Group 2) (Dic exposed group): Six male albino rats were exposed to Dic 10 mg/kg intraperitoneally (I.P) for 28 days [28] with certain modifications, (Group 3) (Caf exposed group): Six male albino rats were exposed to Caf only (15 mg/kg orally) for 28 days [29] with certain modifications; (Groups 4) (Dic + Caf exposed group): Six male albino rats were exposed to Dic (10 mg/kg, i.p) + Caf (15 mg/kg, orally) for 28 days [28, 29].

### Samples handling procedures

At the end of the experimental design, rats’ body weight was first measured on day 0 and then on day 29 using automatic balance. A blood sample was taken from each rat retro-orbital vein followed by centrifugation for 5–10 min at 4℃ /3500 rpm. Serum was stored at -20℃ till being used. Subjected rats were killed by cervical dislocation followed by rapid isolation of the liver and kidney. Each isolated liver and kidney was initially weighted and then prepared for histopathology and biological studies. From each rat, part of the liver and kidney was directly fixed in formalin 10% and then being transferred to ethanol. After sectioning, the separated liver and kidney parts were stained with Hematoxylin &Eosin (H&E) for histopathology studies. While the other parts of the liver and kidney were homogenized in phosphate buffer saline (PBS) according to manufacturer instructions. Isolated supernatant of liver and kidney tissue homogenate was stored till being used. On the other hand, Femurs from each rat were aseptically Isolated and placed in DMEM (Dulbecco’s Modified Eagle Medium). Isolated cells were centrifuged and then fixed using Carnoy’s fixative (3 methanol: 1 acetic acid). Slides were prepared using flame-drying followed by being stained using buffered Giemsa (pH = 6.8). Several metaphase spreads per animal were analyzed for chromosomal aberrations [30, 31].

### Assessment of serum hematology and biological parameters

The assessment of biological parameters in serum was conducted including Hemoglobin [32], Red blood cells (RBCs) [33], and Hematocrit (HCT %) [33]. Additionally, serum AST, ALT, creatinine, and urea levels were determined using available commercial kits purchased from Biodiagnostic, Cairo, Egypt.

### Assessment of tissue biological parameters

Lipid peroxidation content (LPO) was measured using thiobarbituric acid procedure. The resulted chromogen was extracted by using n-butyl alcohol and detected at 532 nm. [34, 35]. Glutathione peroxidases (GPx) was estimated based on the ability of the enzyme to convert glutathione to oxidized glutathione compound. Then the remained glutathione reduces 2-nitrobenzoic acid to form a yellow colored complex measured at 412 nm. GPx activity is indirectly proportional to the degree of color intensity [36, 37]. Meanwhile, nitric Oxide (NO) was estimated using griess reaction where the colored formed product was detected at 540 nm [38], and TNF-α was detected using Elisa kit purchased from (MyBioSource, USA). All these parameters were all detected in the prepared supernatant from liver and kidney homogenate according to the mentioned references and kit instructions.

### Histopathology

The isolated liver and kidney parts were mainly fixed in 10% formalin followed by being transferred to ethanol. After cryosectioning, the separated liver and kidney parts were stained with Hematoxylin and Eosin (H&E) then directly being examined under the light microscope [39].

### Statistical analysis

The obtained data were expressed in the form of Mean ± SD using SPSS 18 and GraphPad Prism 5.0 software. The variance between groups was analyzed using One-Way ANOVA followed by multiple-group comparisons using the Dunnett’s test. Results were considered significant at *p* < 0.05. Graphs were illustrated using GraphPad Prism 5.0 software.

## Results

### Effects of diclofenac administration on organs and body weight

Illustrated Table 1 showed that the daily administration of Caf (group3) resulted in a non-significant difference in the measured whole body weight in addition to kidney and liver body organs weight when compared with the control group (group1) (*p* > 0.05). On the other hand, Table 1 revealed that the administration of Dic (group2) resulted in a significant reduction in the weight of isolated liver and kidney from each rat associated with a relevant significant increase in the whole body weight when compared with the control group (group1). The obtained data also demonstrated that the daily administration of Dic associated with the daily Caf administration for 28 days attenuated the damaging effects of Dic on organs and body weight. Whereas, an observed decrease in whole body weight in addition to significant improvement in organs weight was observed in (group4) (Dic + Caf exposed group) when compared with other subjected groups 1,2&3 as shown in Table 1 (*p* < 0.05).

**Table 1:** Mitigation effect of caffeine against diclofenac-induced damage on body and organs weight (liver & kidney)

Groups	Organs weight		Body weight (g)
	**Liver (g)**	**Kidney (g)**	
**Ctrl group1**	6.29 + 0.140^a^	1.44 + 0.066^a^	244.1 + 5.05^a^
**Dic exposed group2**	5.11 + 0.143^b^	1.155 + 0.062^b^	279.5 + 6.25^b^
**Caf exposed group3**	6.28 + 0.141^a^	1.428 + 0.035^a^	241.0 + 4.6^a^
**Dic + Caf exposed group4**	5.94 + 0.0899^c^	1.30 + 0.052^c^	253.5 + 7.81^c^

Values are represented in the form of mean ± SD of (*n* = 6 per group) (*p* < 0.05). Same represented small letters indicate non-significant different (*p* > 0.05)

### Caffeine ameliorative effects on Diclofenac-induced hematologic disorders

It was observed that administration of Dic (group2) resulted in a significant decrease in the hematologic parameters including Hb%, RBCs, and HCT% associated with a relevant increase in WBCs count when compared with the control group (group1) (*p* < 0.05) as shown in Table 2. Meanwhile, the administration of Caffeine alone (group 3) resulted in no change and non-significant difference in the hematologic parameters when compared with the control group (group 1) (*p* > 0.05) as shown in Table 2. On the other hand, the group of rats received Dic (10 mg/kg, i.p) + Caf (15 mg/kg, orally) represented as (group 4) displayed a well-marked improvement in Hb%, RBCs, HCT%, and WBCs count when compared with other subjected groups 1, 2 & 3 as shown in Table 2 (*p* < 0.05).

**Table 2:** Mitigation effect of caffeine against diclofenac-induced hematologic disorders

Groups	Hb (%)	RBCs (10^6^mm^2^)	HCT (%)	WBCs (10^9^/L)
**Ctrl group1**	13.015 + 0.213^a^	4.33 + 0.070^a^	38.55 + 1.40^a^	5.33 + 0.480^a^
**Dic exposed group2**	10.81 + 0.131^b^	3.60 + 0.042^b^	32.47 + 0.370^b^	8.15 + 0.339^b^
**Caf exposed group3**	13.067 + 0.260^a^	4.35 + 0.085^a^	39.0 + 0.557^a^	5.4 + 0.141^a^
**Dic + Caf exposed group4**	12.14 + 0.173^c^	4.04 + 0.059c	36.48 + 0.423^c^	6.5 + 0.316^c^

Values are represented in the form of mean ± SD of (*n* = 6 per group) (*p* < 0.05). Same represented small letters indicate non-significant different (*p* > 0.05)

### Ameliorative effect of caffeine on liver and kidney functions in Diclofenac-induced liver and kidney damage

**Fig. 1:**
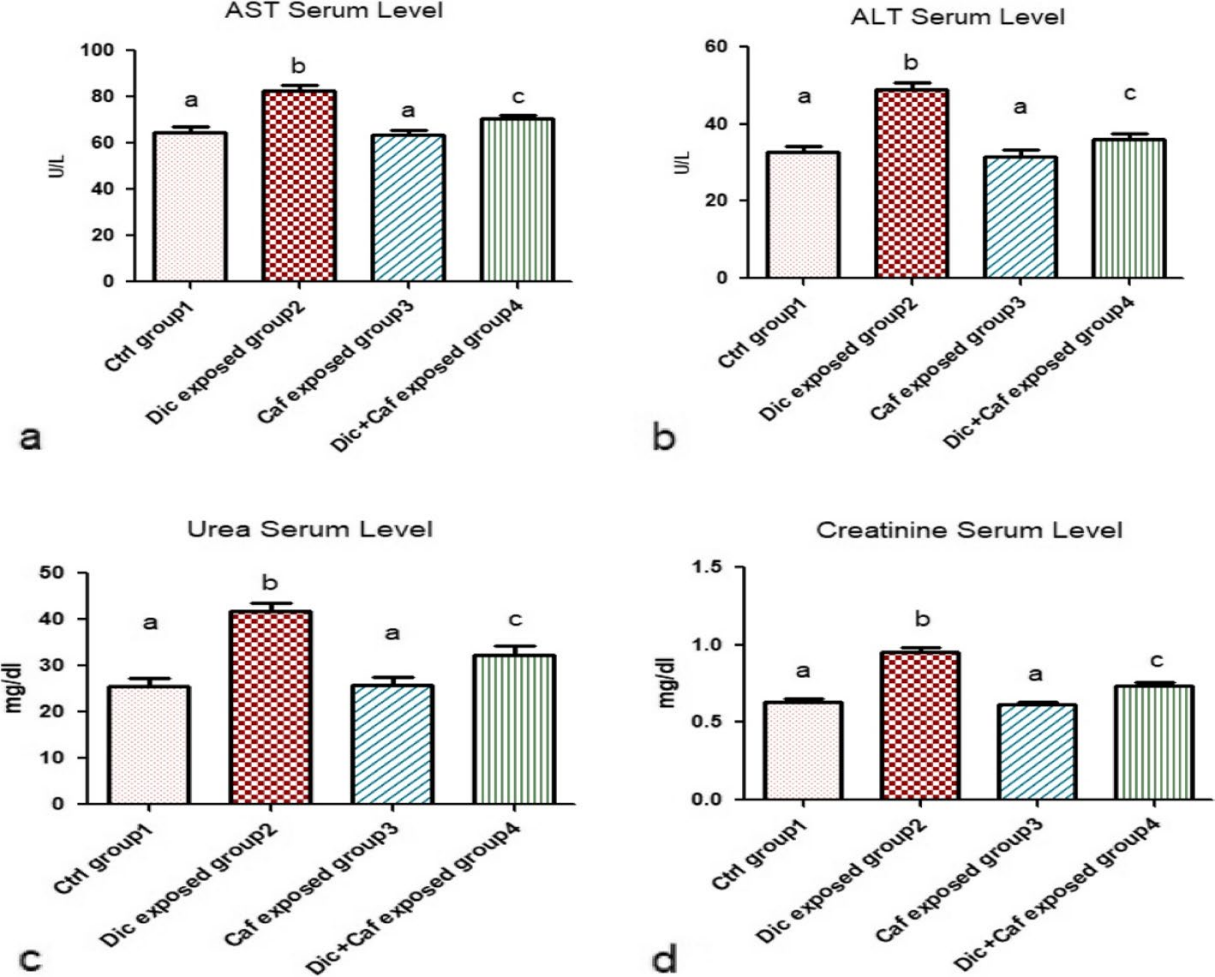
Mitigation effect of Caffeine against Diclofenac-induced damage on liver and kidney functions. **a**, **b** Liver function tests (AST, ALT). **c**, **d** Kidney functions tests (Urea, Creatinine). Values are represented in the form of mean ± SD of (*n* = 6 per group) (*p* < 0.05). Same represented small letters indicate non-significant different (*p* > 0.05)

Illustrated Fig. 1a, b, c and d demonstrated that the effect of Caf sole administration (group3) for 28 days elicited no change and non-significant difference in AST, Alt, urea, and creatinine serum levels when compared with the control group (group I) (*p* > 0.05). Data of the current study also represented in Fig. 1a, b, c and d demonstrated that the daily administration of Dic (group2) resulted in elevated AST, Alt, urea, and creatinine serum levels when compared with the control group (group1) (*p* < 0.05). On the other hand, the administration of Dic (10 mg/kg, i.p) + Caf (15 mg/kg, orally) represented as (group 4) proved that caffeine administration attenuated the undue effect of Dic on liver and kidney function tests represented as AST, Alt, urea, and creatinine serum levels when compared with other subjected groups 1,2&3 as shown in Fig. 1a, b, c and d (*p* < 0.05).

### Antioxidant efficiency and Lipid peroxidation suppressed level by Caffeine administration in Diclofenac-induced liver and kidney damage

**Fig. 2:**
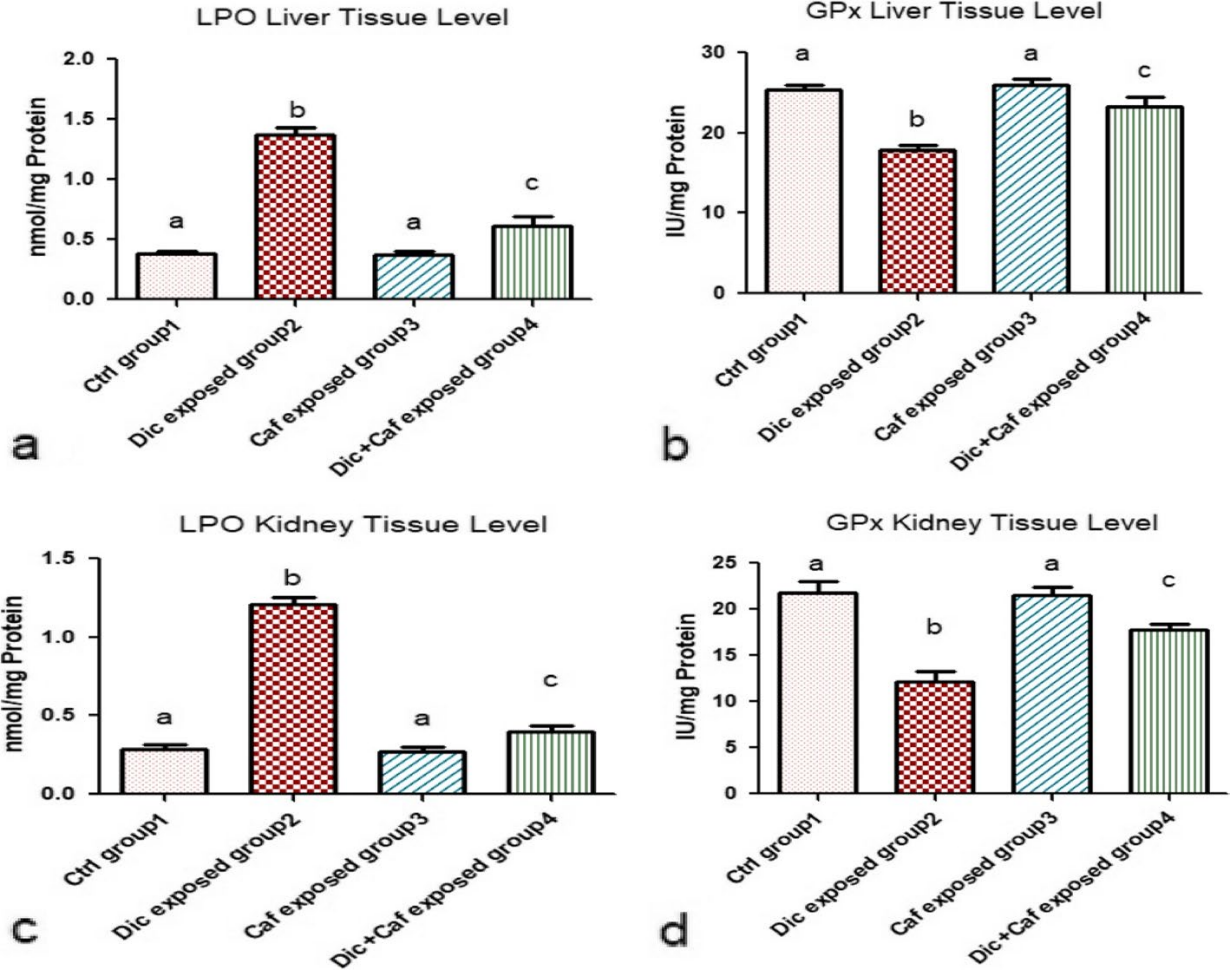
Mitigation effect of Caffeine against Diclofenac-induced damage on oxidative stress in rats’ liver and kidney tissues. **a**, **b** LPO and GPx levels in liver tissue. **c**, **d** LPO and GPx levels in kidney tissue. Values are represented in the form of mean ± SD of (*n* = 6 per group) (*p* < 0.05). Same represented small letters indicate non-significant different (*p* > 0.05)

Observed results revealed that the administration of Dic (group 2) resulted in a significant increase in LPO level in addition to decreased GPx level in both liver and kidney tissue as illustrated in Fig. 2a, b, c and d when compared with the control group (group1) (*p* < 0.05). Whereas, a major significant decrease in LPO level associated with increased GPx liver and kidney tissue level was observed following the administration of Dic (10 mg/kg, i.p) + Caf (15 mg/kg, orally) (group 4) indicating the ameliorative effect of Caf against Dic-induced liver and kidney damage when compared with other subjected groups 1, 2 & 3 as shown in Fig. 2a, b, c and d (*p* < 0.05). on the other hand, Fig. 2a, b, c and d also demonstrated that the administration of Caf alone represented as (group3) revealed a non-significant change in LPO and GPx liver and kidney tissue levels when compared with the control group (group1) (*p* > 0.05).

### Triggered inflammatory markers in Diclofenac-induced liver and kidney damage

**Fig. 3:**
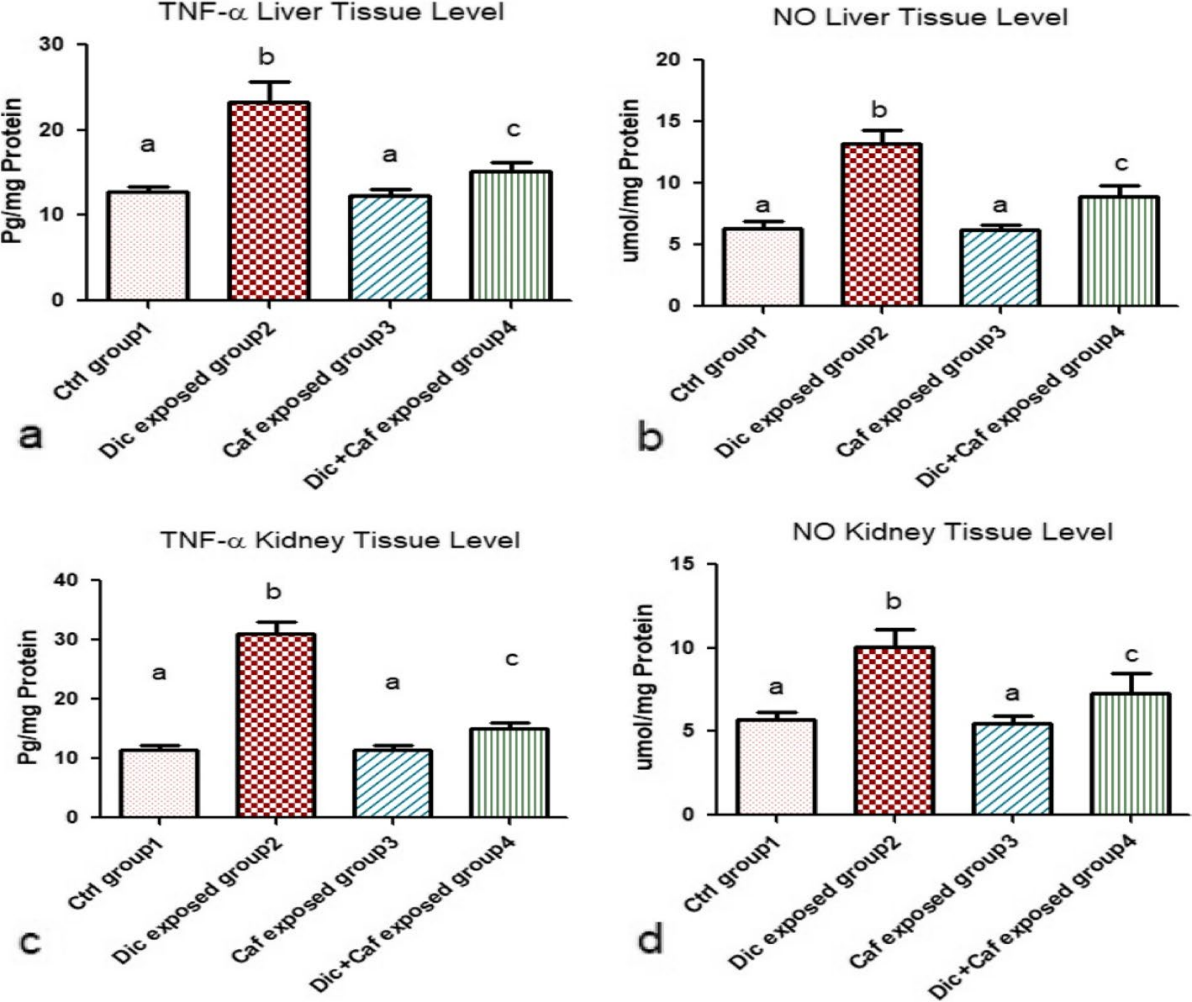
Mitigation effect of Caffeine against Diclofenac-induced damage on inflammatory markers in liver and kidney tissues. **a**, **b** TNF-α and NO levels in Liver tissue. **c**, **d **TNF-α and NO levels in Kidney tissue. Values are represented in the form of mean ± SD of (*n* = 6 per group) (*p *< 0.05). Same represented small letters indicate non-significant different (*p* > 0.05)

As an indicator of the degree of inflammatory damage in liver and kidney tissues, TNF-α and Nitric Oxide (NO) were significantly increased in Dic (group 2) exposed group when compared with the control group (group1) (*p* < 0.05) as shown in Fig. 3a, b, c and d. Meanwhile, these triggered inflammatory levels (TNF-α and NO) were restored after the administration of Dic (10 mg/kg, i.p) + Caf (15 mg/kg, orally) (group 4) indicating the ameliorative effect of Caf against Dic-induced liver and kidney damage when compared with other subjected groups 1,2&3 as shown in Fig. 3a, b, c and d (*p* < 0.05). On the other hand, the administration of Caf alone (group3) revealed a non-significant change in TNF-α and NO liver and kidney tissue levels when compared with the control group (group 1) (*p* > 0.05) as shown in Fig. 3a, b, c and d.

### Caffeine ameliorates chromosomal aberrations in Diclofenac-induced damage on bone marrow:

**Fig. 4:**
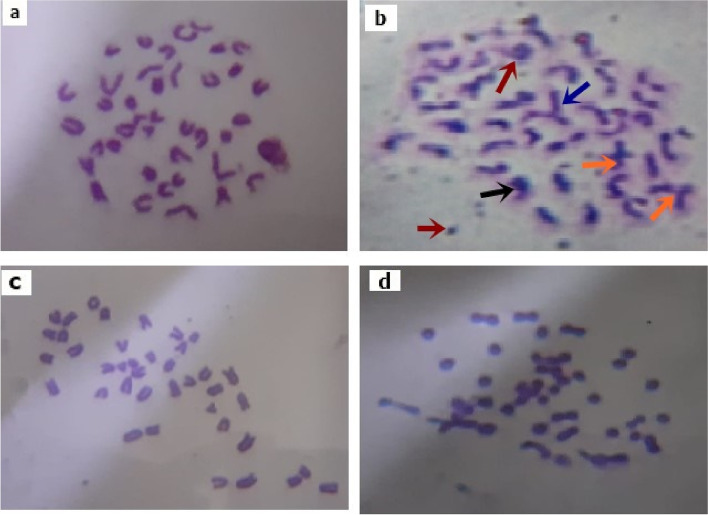
Mitigation effect of Caffeine against Diclofenac-induced damage on bone marrow chromosomal aberrations. **a**, **b**, **c**, **d** Represent the degree of bone marrow chromosomal aberrations in groups (1, 2, 3, 4) respectively. Colored arrows represent chromosomal aberrations structures: (black arrow) dicenteric, (orange arrow) deletion, (red arrow) acenteric fragment, and (blue arrow) translocation in Dic exposed group (group 2)

Illustrated Table 3 and Fig. 4 summarized the degree of bone marrow (BM) chromosomal aberrations following the administration of Dic and/or caffeine where results were represented as follow: chromatid deletions (D), dicentric (D.C), fragment (F), centric separation (CS), ring (R) and polyploidy. These mentioned structural and illustrated types of changes were indicated and identified according to the control Group1. An observed relevant decrease in the degree of chromosomal aberrations was observed following the administration of Dic (10 mg/kg, i.p) + Caf (15 mg/kg, orally) (group 4) when compared with other subjected groups 1, 2 & 3 as illustrated in Table 3 and Fig. 4 (*p* < 0.05). Meanwhile, the degree of total chromosomal aberrations was significantly increased following the administration of Diclofenac (group 2) indicating the degree of bone marrow damage following the daily exposure to Diclofenac (*p* < 0.05). Meanwhile, the sole caffeine administration (group 3) revealed normal chromosomal aberrations level when compared with the control group1 (*p* > 0.05) as illustrated in Table 3 and Fig. 4.

**Table 3:** Mitigation effect of caffeine against diclofenac-induced bone marrow chromosomal aberrations in male albino rats

Groups	Chromosomal aberrations						Total No of aberration	Average No of aberration
	**R**	**D.C**	**F**	**C.S**	**D**	**polyploidy**		
**Ctrl group1**	3	0	0	0	4	1	8	1.0 0.34^a^
**Dic exposed group2**	68	16	55	52	72	25	288	47.21 ± 4.10^b^
**Caf exposed group3**	1	0	3	0	4	2	10	1.17 ± 0.40^a^
**Dic + Caf exposed group4**	2	3	1	7	3	5	21	5.12 ± 0.92^c^

Chromosomal aberration structures represented as: Dicentric (D.C), fragment (F), centric separation (C.S), deletion (D), and polyploidy. An observed induced chromosomal aberrations was detected in Dic exposed group (group 2) when compared with groups (1,3 and 4) (*p* < 0.05)

### Histopathological liver and kidney tissue examination

**Fig. 5:**
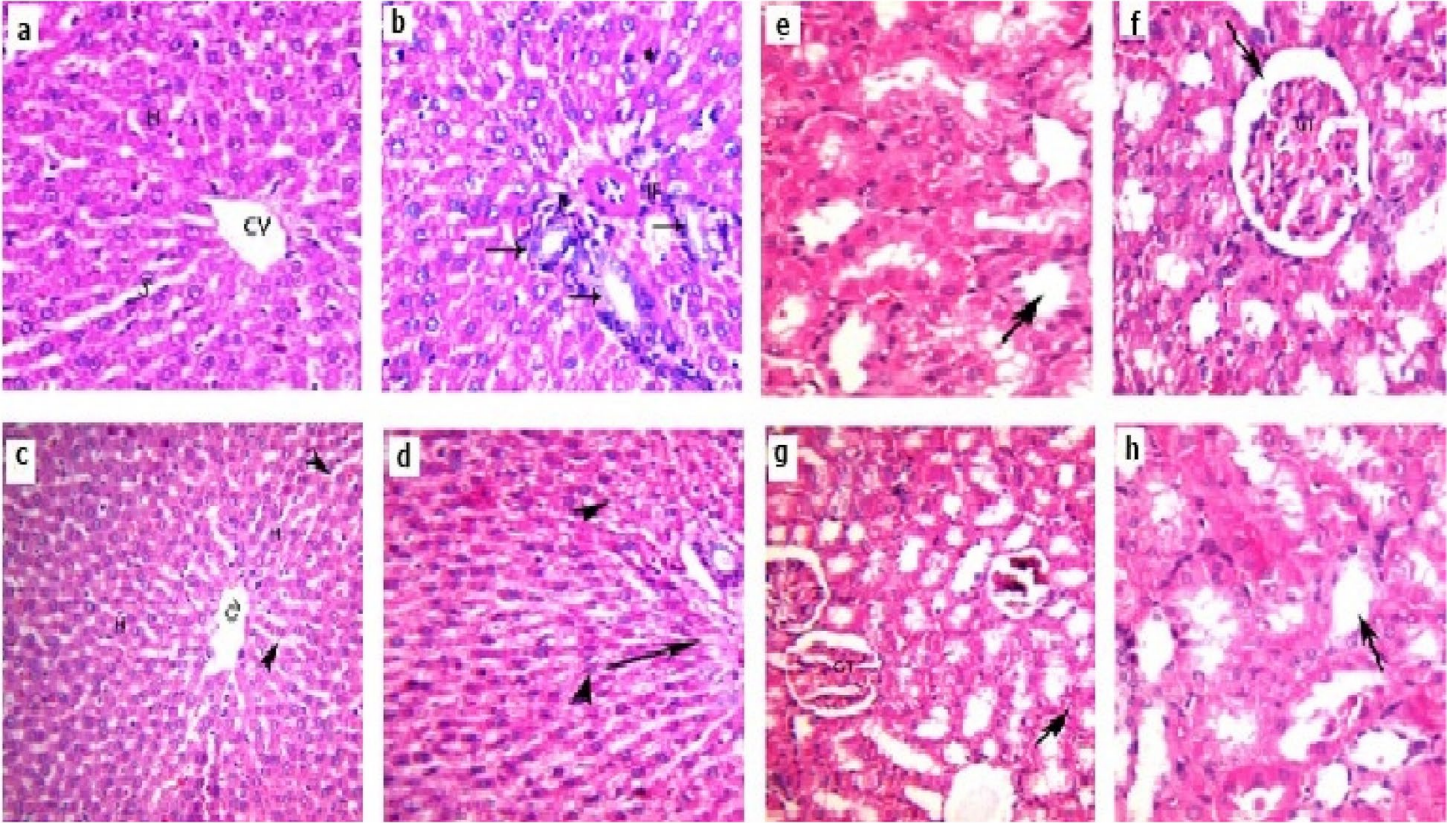
Histopathological illustrations of liver and kidney sections stained in Hematoxylin &Eosin (100x). **a**, **e** Photomicrograph of the control group (group1) intact liver and kidney structures respectively. **b**,** f ** Photomicrograph of Dic exposed liver and kidney damages respectively (group2) indicating constricted portal area with proliferated bile duct (arrow), periportal inflammation (IF) and proliferated van Kupfer cells along with showing glomerular tuft (GT) with dilated congested blood capillaries, and dilated bowman’s space (Arrow). **c**, **g** Photomicrograph of Caf exposed liver and kidney tissues respectively (group3) showing normal intact liver and kidney structure when compared with the control group (group1). **d**,** h** photomicrograph of Dic (10 mg/kg, i.p) + Caf (15 mg/kg, orally) exposed liver and kidney sections respectively (group4) showing constricted portal area (arrow) with very mild vacuolated hepatocytes (head of arrow) and slight aggregation of inflammatory cells in lobulated glomerular tuft (GT) and convoluted tubules in addition to slight cytoplasmic atrophy (Arrow)

No histopathological alterations with intact characterized normal liver and kidney structures were detected in the control (group 1) as shown in Fig. 5a and e respectively. Meanwhile, severe atrophy, degeneration, inflamed liver and kidney tissues were observed in Dic exposed group (group2) as shown in Fig. 5b and f respectively. Whereas, normal and intact histopathological liver and kidney findings were detected in Caf exposed group (group 3) as shown in Fig. 5c and g respectively. On the other hand, very mild atrophy was detected in liver and kidney tissues following the administration of Dic (10 mg/kg, i.p) + Caf (15 mg/kg, orally) (group 4) as shown in Fig. 5d and h.

## Discussion

The overdose administration of Diclofenac can be considered toxic to all subjects including humans or animals. In the current study, the exposure to daily Dic administration for 28 days resulted in increased body weight, exaggerated inflammatory responses, altered normal hematological parameters, abnormal liver and kidney functions, triggered oxidative stress release, and suppressed antioxidant levels [8, 40, 41]. These observed toxicities can be related to the accumulation of reactive metabolites known as 4’ and 5’-hydroxydiclofenac associated with the very highly reactive benzoquinone imines compound [8, 42]. The accumulation of these compounds mainly results in increased inflammatory and oxidative stress responses in addition to decrease glutathione peroxidase capacity level. Thereby, these altered body mechanism results in suppressed body defense actions and abnormal body functions [8, 19, 42–44].

In agreement with our results, Diclofenac daily administration has been observed to cause a direct liver and kidney injuries in subjected rats [45]. Since AST, ALT, urea, and creatinine contents are considered the main serum biological parameters indicators of hepatic and renal damages; therefore any observed raise in the level of these parameters can be implied as hepatic and renal impairments [45]. The current study demonstrated that the daily exposure to Diclofenac resulted in elevated serum levels of AST, ALT, urea, and creatinine associated with increased LPO, NO, and TNF-α levels of both liver and kidney tissues. All these alterations are associated with modified normal hematological and suppressed antioxidants level. Whereas, these findings are in accordance with [8, 40, 41, 46, 47] who reported the severity of hepatic damages and renal toxicity as a drawback of Diclofenac misuse. Meanwhile, the increased creatinine and urea levels were found to be mainly controlled by the GFR (glomerular filtration rate). Thereby, any detected alteration in GFR can be directly correlated with elevated creatinine and urea serum levels accompanied with water retention symptoms [46, 48]. Additionally, these detected alterations are considered major signs of kidney atrophy and necrosis in addition to being considered predisposing factors for renal failure especially in the long term. On the other hand, it is suggested that elevated AST and ALT serum levels may indicate hepatotoxic tissue membrane damage due to the leakage of these enzymes to the systemic circulation [41]. Meanwhile, the increased LPO, and NO liver-kidney tissue levels can be related to decreased antioxidant capacity and free radical scavenging activity [49]. These elevated oxidative stress factors are suggested to activate various transcription factors leading to triggered inflammatory cytokines release such as TNF-α. The elevated TNF-α level in hepatic and renal tissues is suggested to be the main cascading factor for the observed hepatic and renal tissue atrophy in the histopathological studies. Additionally, chromosomal aberrations cytogenetic marker which is the most validated indicator for detecting the degree of DNA damage was significantly induced following Dic administration. These altered chromosomal aberrations may be due to damaged DNA, DNA synthesis inhibition, and topoisomerase II suppression [49].

Caffeine is a compound rich in photochemical derivatives including triterpenes, trigonelline, melanoidins, and flavonoids [50]. The antioxidant and anti-inflammatory capacity can be related to Caffeine related compounds [51], thus the administration of Caffeine significantly reduced the elevated hepatic and renal parameters in Dic exposed rats. Interestingly, the detected ameliorative and protective effect following the administration of Caffeine in Dic exposed group (group4) and in the sole Caffeine exposed rats (group3) on the liver and kidney tissues indicate that Caf has the ability to eliminate ROS and scavenger free radicals due to its efficient antioxidant and anti-inflammatory actions [52, 53]. In accordance with our results, [54] reported that Caffeine consumption activate antioxidant response elements (AREs) which induce the cellular antioxidant system expression [54, 55]. ARE proteins are vital part of the antioxidant-anti-inflammatory system that are responsible for protecting the body by neutralizing the release of free radicals and oxidizing dexterous agents. Whereas, these elevated AREs are suggested to be responsible for regulating chromosomal aberration and DNA damage [54, 55]. Thereby, Caffeine consumption decrease chromosome aberrations following the administration of Diclofenac in rats. Additionally, the anti-inflammatory properties of Caffeine can be related to its suppression ability against COX, NO, and LPO resulting in the hindered release of IL-6, nuclear factor-κB (NF-κB) and TNF-α [53, 54]. On the other hand, the beneficial activity of Caffeine against Dic toxicity on the liver and kidney could be also attributed to its ability in enhancing GPx release level. Meanwhile, the histopathological findings from both the liver and kidney tissues in the current study revealed severe lesions and atrophy following Dic administration. On the other hand, the Caffeine exposed groups demonstrated highly intact hepatic and renal architecture. Thus, indicating the hepato-renal protective efficiency of Caffeine by maintaining normal functions and structural integrity of the liver and kidney against NSAIDs toxicity. Whereas, this observed efficacy can be related to the following caffeine-mediated mechanisms: antioxidant, anti-inflammatory, DNA damage inhibitor, and diuretic potentials [56].

Several previously conducted epidemiological studies have reported the direct link between Dic misuse and hepatic/renal damages in addition to highlighting the ability of caffeine to alleviate or hinder these Dic-induced damages. Owumi, Dim [28] and Elshopakey, Elazab [45] reported in line with our findings that prolonged chronic intake of Dic enhance cellular oxidative damage and inflammatory cytokines release including ROS, NO, TNF-α, and NF-kB resulting in severe deteriorations on the liver and kidney. Herein, we provide a clear evidence in agreement with previously reported results that the treatment with caffeine significantly alleviated the severe degree of liver and kidney damages induced by Dic administration [20, 21, 53, 57, 58]. This indicates the efficiency of daily caffeine administration on protecting the liver and kidney form Dic deleterious effect on prolong use due to its antioxidant, anti-inflammatory, diuretic potentials, and protective effect against DNA damage [20, 21, 28, 45, 53, 57, 58].

### Study limitations

This study was only conducted on male rats where this can be considered a kind of limitations as female hormones might augments the alleviating actions of caffeine against Dic-induced liver and kidney damages [59]. Previous studies have reported that gender differences may affect caffeine metabolism and physiological responses to daily caffeine administration [59]. Thus, further studies are required to study the effects of female sex hormones against daily caffeine administration.

## Conclusion

The findings of the current study displayed that the daily exposure to Diclofenac elicited severe hepatic and renal damage in rats which can be related to triggered oxidative stress, DNA damages and inflammatory cytokines release. Caffeine is highly rich in several photochemical derivatives including flavonoids, triterpenes, and polyphones where its beneficial actions can be attributed to its several constituents. Thus, our observed results indicated that daily Caffeine administration relieved Diclofenac-induced hepatic and renal damages by enhancing the overall antioxidant capacity status, and suppressing pro-inflammatory cytokines release, inhibiting chromosomal aberrations, and adjusting hematological abnormalities. Thereby it can be concluded that the daily administration of Caffeine can help to alleviate drugs and chemicals-induced hepatic and renal damage via various mechanisms indicating the importance of developing novel pharmaceutically designed drugs containing caffeine compounds for it various therapeutic potentials. Thus, it is recommended to subsequently consider Caffeine to be further assessed in major clinical trials for reducing hepatic and renal induced damages as a drawback of the daily administration of OTC drugs such as Diclofenac to be more ascertain of its efficacy and efficiency clinically.

## Data Availability

The obtained data analyzed during the current study are available from the corresponding author on reasonable request.

## Abbreviations

Dic: Diclofenac
NSAID: Non-steroidal anti-inflammatory drug
ROS: Reactive Oxygen Species
DMEM: Dulbecco’s Modified Eagle Medium
LPO: Lipid peroxidation
TNF-α: Tumor Necrosis Factor-α
AST: Aspartate Aminotransferase
ALT: Alanine Aminotransferase
NO: Nitric Oxide
